# Mechanistic Insights and Future Directions for Enfortumab Vedotin in Urothelial Carcinoma: Highlights from the 10th Annual Leo & Anne Albert Institute for Bladder Cancer Care and Research Symposium

**DOI:** 10.3390/curroncol32050278

**Published:** 2025-05-14

**Authors:** Catherine C. Fahey, Sean Clark-Garvey, Sima Porten, Ashish M. Kamat, Thomas W. Flaig, John A. Taylor, William Y. Kim, Matthew I. Milowsky

**Affiliations:** 1 Lineberger Comprehensive Cancer Center, University of North Carolina, Chapel Hill, NC 27599, USA; 2 Department of Urology, University of California, San Francisco, CA 94143, USA; 3 Department of Urology, MD Anderson Cancer Center, Houston, TX 77030, USA; 4 Department of Medicine, University of Colorado, Aurora, CO 80045, USA; 5 Department of Urology, University of Kansas, Kansas City, KS 66160, USA

**Keywords:** urothelial carcinoma, antibody drug conjugate, immunotherapy, enfortumab vedotin, pembrolizumab, biomarkers

## Abstract

Enfortumab vedotin (EV) in combination with pembrolizumab (P) has led to a new paradigm for the treatment of metastatic urothelial carcinoma (mUC). Since the presentation of the results of the EV-302 trial at the European Society of Medical Oncology 2023 annual meeting, the entire treatment landscape for mUC has been upended. At the 2024 Albert Symposium, we reviewed ongoing research investigating predictive biomarkers for EV response and resistance as well as clinical trials exploring the potential role for EV in different clinical disease states including non-muscle invasive and muscle-invasive disease.

## 1. Current Role for EV in mUC

In EV-301, a phase 3 randomized controlled trial comparing EV to investigator’s choice chemotherapy (docetaxel, paclitaxel, or vinflunine) for patients with mUC who had progressed after platinum-based chemotherapy and immune checkpoint inhibition, EV demonstrated a significant improvement in progression free survival (PFS) compared with chemotherapy (median PFS, 5.55 months versus 3.71 months; HR, 0.62; 95% confidence interval (CI), 0.51–0.75) [1]. EV also improved overall survival (OS) (median, 12.88 months versus 8.97 months; HR, 0.70; 95% CI 0.56–0.89). Enfortumab vedotin in combination with pembrolizumab (EV + P) was studied in previously untreated patients who were not eligible for cisplatin-based chemotherapy in Cohort K of the EV-103 study, a phase Ib/II study of EV monotherapy or in combination with pembrolizumab [2]. In this cohort, the overall response rate (ORR) was 64.5% for EV + P, with 65.4% of patients who responded maintaining a response at 12 months. These results were highly encouraging for the activity of this combination in mUC.

In the EV-302 trial, patients with untreated locally advanced or mUC were randomized to treatment with either EV + P versus standard of care platinum doublet chemotherapy [3]. The trial was powered by dual primary endpoints of PFS and OS. EV + P improved median PFS from 6.3 months with chemotherapy to 12.5 months (HR, 0.45; 95% CI 0.38–0.54). Median OS was also improved from 16.1 months with chemotherapy to 31.5 months (HR, 0.47; 95% CI 0.38–0.58) with EV + P. ORR was significantly improved from 44.4% with chemotherapy to 67.7% with EV + P, including a 29.1% complete response rate. At the ASCO GU 2025 meeting, the results after an additional 12 months of follow-up (total follow-up of 29.1 months) for EV-302 were presented [4]. In this updated analysis, the median PFS remained 12.5 months, while the median OS was 33.8 months. The median duration of response was 23.3 months in the EV + P treated patients, compared with 7.0 months in the control arm. There was a 30.4% clinical complete response rate.

These results were unprecedented in the treatment of mUC and have rapidly become the new standard of care in the first-line setting. The 2024 EAU, 2024 ESMO, and 2024 NCCN guidelines recommend EV + P for all patients with mUC in the first-line setting who are eligible for combination therapy, reserving chemotherapy only for those deemed ineligible for EV, those who lack access to EV, or those ineligible for an immune checkpoint inhibitor [5,6,7].

## 2. EV Mechanism of Action

**Figure 1:**
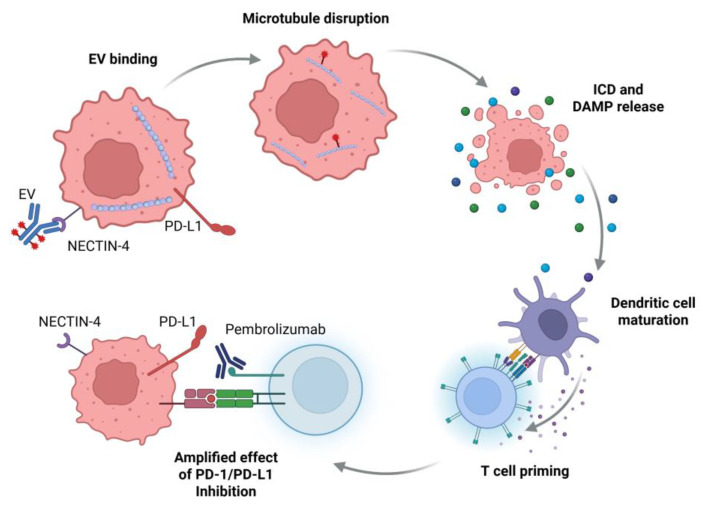
Potential mechanism of action of EV + P and ICD.

EV is a NECTIN4-directed antibody drug conjugate (ADC), with a chemotherapy payload of the microtubule-disrupting agent monomethyl auristatin E (MMAE). NECTIN4 overexpression is associated with increased rates of proliferation, invasion, and epithelial–mesenchymal transition (EMT) [8]. NECTIN4 is highly expressed in bladder cancer, making it an attractive target [9]. Preclinical models have suggested that the combination of EV with a programmed cell death-1 (PD-1) inhibitor leads to enhanced antitumor activity and lasting antitumor immunity [10]. There is evidence that immunogenic cell death (ICD), a regulated mechanism of cellular death that is sufficient to activate an adaptive immune response in an immunocompetent host [11], contributes to the response to EV [10]. ICD occurs through the release of Damage-associated molecular patterns (DAMPs), endogenous molecules that are exposed or released when a cell undergoes stress, injury or death. When exposed, DAMPs are able to bind to receptors on immune cells resulting in inflammatory signaling which leads to dendritic cell maturation and T cell priming [12]. MMAE binds to microtubules and causes microtubule dysregulation [13], resulting in endoplasmic reticulum (ER) stress [14]. EV induces ICD through this microtubule disruption and ER stress [10]. The ability of MMAE to induce ICD suggests a potential for synergy with immune checkpoint inhibition and MMAE conjugated ADCs, such as EV (Figure 1). In addition to EV-302, other studies of MMAE-based ADCs have demonstrated encouragingly high response rates when given in conjunction with immune checkpoint inhibition, even among heavily pre-treated populations [15,16,17].

This mechanism of action for ICD with EV is further supported by the relative success of MMAE-based ADCs compared to other payloads such as camptothecin in mUC. Disitamab vedotin is a HER2-directed ADC with a MMAE payload that has been studied in combination with toripalimab, a PD-1 targeting agent, in a phase 1b/2 study [18]. In this study, HER-2 unselected patients with locally advanced/mUC had an ORR to disitamab vedotin + toripalimab of 73.2%. Conversely, the HER2-directed ADC traztuzumab deruxtecan, which uses a camptothecin payload, only had a 36.7% ORR among patients who were selected to be HER2+ by immunohistochemistry when used in combination with nivolumab [19]. These studies cannot be directly compared, as the disitamab vedotin study included treatment naïve patients, while the trastuzumab deruxtecan study only included patients who had progressed on prior platinum-based therapy. Indeed, EV only had a marginally higher ORR of 40.6% when studied in the platinum refractory population; however, this response was for EV monotherapy, and the study enrolled all patients, not biomarker-selected patients as in the trastuzumab deruxtecan study [1].

## 3. Predictive Biomarkers for EV Response

Despite the overwhelming success of EV + P in the frontline treatment of mUC, there remain patients who do have primary disease progression or progress on treatment. In EV-302, 8.7% of patients had progressive disease as a best response, while 56.1% had progression within 18 months [3]. Identification of biomarkers of both response and resistance will allow better patient selection. There is some evidence that the cutaneous toxicity seen with EV is associated with response [20,21]. In a retrospective study of 51 patients treated with more than one dose of EV, 48% had a cutaneous toxicity [20]. Radiographic response for those with cutaneous toxicity was 58%; for those without, the response rate was 24%. In a follow-up analysis, cutaneous toxicity correlated with improved OS (HR, 0.48; CI 0.25–0.9); however, median PFS was not significantly longer in multivariate analysis [21]. The cutaneous toxicity is likely related to NECTIN4 expression in the skin, including the epidermis and the epithelium of sweat glands and hair follicles [22]. It remains unclear as to the mechanism behind the potential relationship between the cutaneous toxicity and response. In the study examining radiographic response described above, the authors noted that, of four patients who developed the highest grade toxicity, two were treated at the maximum dose [20]. Cutaneous toxicity is also more likely to occur in the first cycle and may be managed with dose reduction in the following cycles. As such, high initial drug exposure may be the driver of cutaneous toxicity and response. Alternative hypotheses could include germline genetic polymorphisms that alter EV metabolism or modulate NECTIN4 levels in the epidermis.

*NECTIN4* mRNA expression has been shown to be enriched in luminal bladder tumors relative to basal bladder tumors [23]. Functional, in vitro studies in bladder cancer cell lines showed that knockdown of *NECTIN4* promotes resistance to EV, validating that NECTIN4 is required for response to EV. The majority of the emerging biomarker data relate to protein expression or genomic amplification of the target of EV, NECTIN4 (also known as *PVRL4*). In an analysis of samples from the EV-101 phase I trial of EV monotherapy, 96.7% of cases demonstrated high total NECTIN4 expression (H-score > 150) by immunohistochemistry (IHC) using the proprietary M22-321b41.1 anti-NECTIN4 antibody clone [24]. NECTIN4 expression was therefore not thought to be a useful biomarker of response. Indeed, in the analysis of Cohort K of the EV-103 study, in which cisplatin-ineligible patients were randomized to EV or EV + P, NECTIN4 expression at baseline did not significantly differ between responders and non-responders [2]. However, since ADCs only bind proteins expressed on the cell surface, Eckstein and colleagues examined the relationship between membranous NECTIN4 expression (using a commercially available antibody clone: EPR15613-68 (Abcam, Cambridge, MA, USA)) and EV response [25]. They found that 19.7% of primary tumors were NECTIN4 negative, 28.4% weak, 26.3% moderate, and 25.5% strong with respect to membranous expression. When correlated with response, membranous NECTIN4 negative and NECTIN4 weak staining was associated with a reduced PFS and a 4-fold increased risk of progression on EV compared with moderate/strong NECTIN4 expression. Moreover, they examined NECTIN4 expression in a subset of patients with paired metastases and found that nearly 60% of metastases had decreased membranous NECTIN4 expression compared to primary tumors. This discordance may play a role in both primary resistance and subsequent disease progression when treated with EV.

Finally, up to 26% of mUC demonstrate amplification of NECTIN4 by FISH [26]. Importantly, *NECTIN4* amplification status (amplified or non-amplified) is conserved between primary tumors and matched metastases in 93% of the examined cases. This amplification is highly associated with response, as a striking 96% of patients with *NECTIN4* amplification had an objective response to EV, compared with 32% of patients within the non-amplified subgroup. *NECTIN4* amplification was also associated with 90% PFS at 12 months in the amplified subgroup versus 41% of the non-amplified subgroup. The median OS was not reached for *NECTIN4* amplified patients and was 8.8 months for non-amplified patients.

## 4. Potential for EV in Other Clinical Disease States: MIBC

Based on the overwhelming success of EV and EV + P in the metastatic setting, ongoing clinical trials are evaluating EV, EV-P as well as other EV combinations in earlier clinical disease states. EV-103 Cohort H was the first trial to examine neoadjuvant EV in patients with muscle-invasive bladder cancer (MIBC) [27]. In this study, cisplatin ineligible patients with MIBC who were fit for surgery were treated with neoadjuvant single-agent EV for 3 cycles followed by radical cystectomy with lymph node dissection. A pathologic complete response (pCR) was seen in 36.4% and pathologic downstaging (pDS, defined as ≤ypT1 N0 at cystectomy) occurred in 50% of patients. There was no delay in surgery due to EV-related adverse events. The event-free survival rate at 12 months was 76.4%. EV-103 also had a perioperative arm, cohort L. Patients in cohort L were treated with neoadjuvant single-agent EV for three cycles followed by radical cystectomy with lymph node dissection, followed by six cycles of adjuvant EV. The initial results from the neoadjuvant/RC + PLND phase and 30 days post-surgery were presented at ESMO 2023 [28]. In that cohort, 34% of patients had a pCR and 42% had pDS.

EV-P is also being studied in the perioperative setting (Table 1). The KEYNOTE-B15/EV-304 (NCT04700124) study has enrolled cisplatin-eligible patients with MIBC, and randomized to either EV + P or gemcitabine plus cisplatin, both for four neoadjuvant cycles [29]. Patients undergo radical cystectomy and those who received EV + P continue EV for an additional 5 cycles and pembrolizumab for an additional 13 cycles. A limitation of this study is the lack of immunotherapy in the chemotherapy arm based on the recently reported NIAGARA study which showed a significant benefit for the addition of durvalumab to gemcitabine plus cisplatin in the perioperative setting [30]. Additionally, nivolumab is now FDA approved for the adjuvant treatment of MIBC, based on the results of the CheckMate-274 trial [31]. This trial compared one year of treatment with adjuvant nivolumab to placebo for patients with high-risk MIBC after surgery. The DFS at 6 months was 74.9% with nivolumab and 60.3% with placebo (HR, 0.70; 98.22% CI 0.55–0.90). Given the increasing integration of both neoadjuvant and adjuvant immunotherapy, trials will need to adapt to ensure the most relevant control arms are being utilized.

The KEYNOTE-905/EV-303 (NCT03924895) trial also aims to address the utility of perioperative EV [33]. In this study, cisplatin ineligible patients are randomized to receive perioperative pembrolizumab versus perioperative EV + P versus no adjuvant/neoadjuvant treatment. A key differentiator between KEYNOTE-B15 and KEYNOTE-905 is the inclusion criteria of cisplatin eligible versus cisplatin ineligible patients.

Another ongoing trial, VOLGA (NCT04960709), will also examine perioperative treatment for cisplatin ineligible patients with MIBC [35]. This trial evaluates EV with dual checkpoint inhibition: durvalumab in combination with tremelimumab, a cytotoxic T-lymphocyte antigen 4 (CTLA-4) inhibitor. Patients are randomized to perioperative triplet therapy with durvalumab and tremelimumab plus EV versus perioperative doublet therapy with durvalimumab plus EV versus no neoadjuvant treatment. The EV-ECLIPSE trial will examine the use of perioperative treatment for patients with node positive disease [37]. INTerpath-005 will use a perioperative strategy to examine the addition of V940—mRNA-4157 to EV [38].

EV + P will also be studied in the setting of trimodality bladder preservation. An upcoming phase Ib/II study (NCT06470282) will examine EV + P in MIBC patients who are unable or unwilling to undergo radical cystectomy, and treat with bladder sparing trimodal therapy including maximum transurethral resection of bladder tumor followed by combination chemotherapy and radiation [39]. The primary outcomes of this trial will be the safety of combining EV-P with radiation and the clinical complete response rate in the phase II cohort.

## 5. Potential for EV in Other Clinical Disease States: Non-Muscle Invasive Bladder Cancer and Upper Tract Urothelial Carcinoma

EV is also being explored in the non-muscle invasive bladder cancer (NMIBC) setting. The EV-104 study (NCT05014139) is a phase 1 trial examining intravesical EV for patients with BCG unresponsive NMIBC [40]. The standard of care for these patients is cystectomy, although pembrolizumab [41], nadofaragene firadenovec-vncg [42] and nogapendekin alfa inbakicept-pmln + BCG [43] are FDA approved in this clinical setting [44,45,46]. In EV-104, intravesical EV is administered weekly for a 6 week induction followed by 9 monthly doses and evaluated for both safety and efficacy.

The phase 2 NEPTUNE trial (NCT06356155) examines the use of EV + P perioperatively for high-grade localized/locally advanced upper tract urothelial cancer who are deemed eligible for curative-intent surgery [47]. It will enroll patients who are eligible for cisplatin and treat with 4 cycles of neoadjuvant EV + P prior to nephroureterectomy or distal ureterectomy followed by 13 cycles of adjuvant pembrolizumab. Another phase 2 trial (NCT05868265) is examining neoadjuvant EV + P for high grade urothelial carcinoma of the upper tract [48]. In this trial, patients will receive three cycles of EV + P prior to definitive surgery, without adjuvant therapy planned.

## 6. Novel NECTIN4 Targeting Agents

In addition to EV, new agents that target NECTIN4 are in development, including several ADCs (Table 2) [49,50]. These ADCs will incorporate various toxins, including MMAE (9MW2821 [51], SYS6002/CRB-701 [52]), exatecan, (LY4101174 [53], BAT8007 [54]), AP052 (ADRX-0706 [55]), camptothecin (LY4052031 [56]), and rezetecan (SHR-A2102 [57]). Advances are occurring in the ADC field with the emergence of conditionally active biologic (CAB) ADCs, which are designed to specifically bind to the target antigen of choice only in the tumor microenvironment [58]. BA3361 [59] is one upcoming CAB ADC targeting NECTIN4 that was recently granted FDA IND clearance [60]. There are also exciting new bicyclic toxin conjugates (BTC) targeting NECTIN4 [49]. BT8009 is a peptide that binds NECTIN4 connected to a MMAE toxin via a cleavable linker [61]. BT7480 expands on this by combining a NECTIN4 targeting bicycle with two CD137 bicycles [62]. BT7480 binds to NECTIN4 and agonizes CD137 on local immune cells to alter the immune microenvironment and target cancer cells. Results from the phase I study of SHR-A2102 were presented at ASCO GU 2025. In this study of 73 patients, there was an ORR of 38.4%, including a response rate of 38.7% in patients who had previously received an ADC [57]. This drug is now moving forward into phase II studies, and into a phase III study in China. There is also excitement regarding proteolysis-targeting chimeras (PROTACs) [63]. PROTACS consist of a protein-binding component linked to an E3 ubiquitin ligase binding component. Binding of the PROTAC leads to protein degradation of the target. Understanding the efficacy, safety, and potential for combination therapy will be key in evaluating new NECTIN4 directed payloads.

## 7. Conclusions

The use of EV and EV-P has revolutionized treatment for patients with mUC, and further work hopes to expand on the current successes and approved indications. While NECTIN4 amplification is a potential biomarker of efficacy, additional work including prospective validation is needed to further develop biomarkers of response and resistance. We eagerly await the results of ongoing studies in earlier clinical disease states that are poised to once again change the treatment landscape for patients with urothelial carcinoma.

## Figures and Tables

**Table 1:** Ongoing perioperative clinical trials of EV in muscle invasive bladder cancer (MIBC).

Trial Name	Patient Eligibility	Neoadjuvant Treatment	Adjuvant Treatment	Primary Endpoint	Planned Completion
KEYNOTE-B15/EV-304 [29] NCT04700124	Cisplatin eligible	EV + P versus gemcitabine + cisplatin	EV + P versus observation	EFS	December 2026 [32]
KEYNOTE-905/EV-303 [33] NCT03924895	Cisplatin ineligible	Pembrolizumab versus EV + P versus none	Pembrolizumab versus EV + P versus none	EFS	December 2027 [34]
VOLGA [35] NCT04960709	Cisplatin ineligible	Durvalumab + Tremelimumab + EV versus Durvalumab + EV versus none	Durvalumab + Tremelimumab versus Durvalumab versus none	EFS	September 2028 [36]
EV-ECLIPSE [37] NCT05239624	Cisplatin eligible and ineligible, lymph node involvement	EV + P	Pembrolizumab	pCR	June 2026 [37]
INTerpath-005 [38] NCT06305767 Perioperative Cohort	Cisplatin ineligible	EV + P + V940	EV + P + V490	AE rate, treatment discontinuation	October 2031 [38]

EV—enfortumab vedotin, EV + P—enfortumab vedotin plus pembrolizumab, pCR—pathologic complete response, EFS—event-free survival, AE—adverse event, V940—mRNA-4157.

**Table 2:** NECTIN4 targeting agents in development.

Drug	Mechanism	Toxin	Company	Status
BT8009	BTC	MMAE	Bicycle Therapeutics, Cambridge, UK	I/II/III
BT7480	BTC		Bicycle Therapeutics, Cambridge, UK	I/II
9MW2821	ADC	MMAE	Mabwell, Shanghai, China	I/II/III
LY4101174	ADC	exatecan	Lilly, Indianapolis, IN, USA	I
BA3361	CAB ADC	MMAE	BioAlta, San Diego, CA, USA	IND
BAT8007	ADC	exatecan	Bio-Thera, Guangzhou, China	I
ADRX-0706	ADC	AP052	Adcentrx Therapeutics, San Diego, CA, USA	I
SYS6002/ CRB-701	ADC	MMAE	Corbus Pharmaceuticals, Norwood, MA, USA	I/II
SHR-A2102	ADC	Rezetecan	Jiangsu HengRui, Lianyungang, China	I/II/III

BTC—bicyclic toxin conjugate, ADC—antibody drug conjugate, CAB—conditionally active biologic, MMAE—Monomethyl auristatin E.

## Abbreviations

The following abbreviations are used in this manuscript:

EV	Enfortumab vedotin
mUC	Metastatic urothelial carcinoma
CI	Confidence Interval
EV + P	Enfortumab vedotin in combination with pembrolizumab
ADC	Antibody drug conjugate
MMAE	Monomethyl auristatin E
EMT	Epithelial–mesenchymal transition
PD-1	Programed cell death-1
ICD	Immunogenic cell death
DAMPs	Damage-associated molecular patterns
OS	Overall Survival
PFS	Progression free survival
ORR	Overall response rate
MIBC	Muscle invasive bladder cancer
pCR	Pathologic complete response
pDS	Pathologic downstaging
EAU	European Association of Urology
ESMO	European Society for Medical Oncology
ASCO	American Society for Clinical Oncology
NMIBC	Non-muscle invasive bladder cancer
CAB	Conditionally active biologic
BTC	Bicyclic toxin conjugate

## References

[1] Powles T., Rosenberg J.E., Sonpavde G.P., Loriot Y., Durán I., Lee J.-L., Matsubara N., Vulsteke C., Castellano D., Wu C. (2021). Enfortumab Vedotin in Previously Treated Advanced Urothelial Carcinoma. N. Engl. J. Med..

[2] O’Donnell P.H., Milowsky M.I., Petrylak D.P., Hoimes C.J., Flaig T.W., Mar N., Moon H.H., Friedlander T.W., McKay R.R., Bilen M.A. (2023). Enfortumab Vedotin with or Without Pembrolizumab in Cisplatin-Ineligible Patients with Previously Untreated Locally Advanced or Metastatic Urothelial Cancer. J. Clin. Oncol..

[3] Powles T., Valderrama B.P., Gupta S., Bedke J., Kikuchi E., Hoffman-Censits J., Iyer G., Vulsteke C., Park S.H., Shin S.J. (2024). Enfortumab Vedotin and Pembrolizumab in Untreated Advanced Urothelial Cancer. N. Engl. J. Med..

[4] Powles T., Van Der Heijden M.S., Loriot Y., Bedke J., Valderrama B.P., Iyer G., Kikuchi E., Hoffman-Censits J., Vulsteke C., Drakaki A. (2025). EV-302: Updated Analysis from the Phase 3 Global Study of Enfortumab Vedotin in Combination with Pembrolizumab (EV+P) vs Chemotherapy (Chemo) in Previously Untreated Locally Advanced or Metastatic Urothelial Carcinoma (La/mUC). J. Clin. Oncol..

[5] EAU Guidelines—Uroweb. https://uroweb.org/guidelines/muscle-invasive-and-metastatic-bladder-cancer.

[6] Flaig T.W., Spiess P.E., Abern M., Agarwal N., Bangs R., Buyyounouski M.K., Chan K., Chang S.S., Chang P., Friedlander T. (2024). NCCN Guidelines^®^ Insights: Bladder Cancer, Version 3.2024: Featured Updates to the NCCN Guidelines. J. Natl. Compr. Canc. Netw..

[7] Powles T., Bellmunt J., Comperat E., Santis M.D., Huddart R., Loriot Y., Necchi A., Valderrama B.P., Ravaud A., Shariat S.F. (2024). ESMO Clinical Practice Guideline Interim Update on First-Line Therapy in Advanced Urothelial Carcinoma. Ann. Oncol..

[8] Bouleftour W., Guillot A., Magne N. (2022). The Anti-Nectin 4: A Promising Tumor Cells Target. A Systematic Review. Mol. Cancer Ther..

[9] Challita-Eid P.M., Satpayev D., Yang P., An Z., Morrison K., Shostak Y., Raitano A., Nadell R., Liu W., Lortie D.R. (2016). Enfortumab Vedotin Antibody–Drug Conjugate Targeting Nectin-4 Is a Highly Potent Therapeutic Agent in Multiple Preclinical Cancer Models. Cancer Res..

[10] Olson D., Younan P., Liu B., Blahnik-Fagan G., Gosink J., Snead K., Tenn E., Hensley K., Sahetya D., Nesterova A. (2022). 1187 Enfortumab Vedotin Induces Immunogenic Cell Death, Elicits Antitumor Immune Memory, and Shows Enhanced Preclinical Activity in Combination with Immune Checkpoint Inhibitors. J. Immunother. Cancer.

[11] Galluzzi L., Vitale I., Warren S., Adjemian S., Agostinis P., Martinez A.B., Chan T.A., Coukos G., Demaria S., Deutsch E. (2020). Consensus Guidelines for the Definition, Detection and Interpretation of Immunogenic Cell Death. J. Immunother. Cancer.

[12] Garg A.D., Galluzzi L., Apetoh L., Baert T., Birge R.B., Bravo-San Pedro J.M., Breckpot K., Brough D., Chaurio R., Cirone M. (2015). Molecular and Translational Classifications of DAMPs in Immunogenic Cell Death. Front. Immunol..

[13] Best R.L., LaPointe N.E., Azarenko O., Miller H., Genualdi C., Chih S., Shen B.-Q., Jordan M.A., Wilson L., Feinstein S.C. (2021). Microtubule and Tubulin Binding and Regulation of Microtubule Dynamics by the Antibody Drug Conjugate (ADC) Payload, Monomethyl Auristatin E (MMAE): Mechanistic Insights into MMAE ADC Peripheral Neuropathy. Toxicol. Appl. Pharmacol..

[14] Klussman K., Tenn E.-M., Higgins S., Mazahreh R., Snead K., Hamilton J., Grogan B., Sigurjonsson J., Cao A., Gardai S. (2020). 618 Vedotin ADCs Induce ER Stress and Elicit Hallmarks of ICD across Multiple Cancer Indications. J. Immunother. Cancer.

[15] Wang Y., Gong J., Wang A., Wei J., Peng Z., Wang X., Zhou J., Qi C., Liu D., Li J. (2024). Disitamab Vedotin (RC48) plus Toripalimab for HER2-Expressing Advanced Gastric or Gastroesophageal Junction and Other Solid Tumours: A Multicentre, Open Label, Dose Escalation and Expansion Phase 1 Trial. eClinicalMedicine.

[16] Lee H., Flinn I.W., Melear J., Ramchandren R., Friedman J., Burke J.M., Linhares Y., Gonzales P.A., Raval M., Chintapatla R. (2022). Brentuximab Vedotin, Nivolumab, Doxorubicin, and Dacarbazine (AN+AD) for Advanced Stage Classic Hodgkin Lymphoma: Updated Efficacy and Safety Results from the Single-Arm Phase 2 Study (SGN35-027 Part B). Blood.

[17] Vergote I., Van Nieuwenhuysen E., O’Cearbhaill R.E., Westermann A., Lorusso D., Ghamande S., Collins D.C., Banerjee S., Mathews C.A., Gennigens C. (2023). Tisotumab Vedotin in Combination With Carboplatin, Pembrolizumab, or Bevacizumab in Recurrent or Metastatic Cervical Cancer: Results From the innovaTV 205/GOG-3024/ENGOT-Cx8 Study. J. Clin. Oncol..

[18] Zhou L., Yang K.W., Zhang S., Yan X.Q., Li S.M., Xu H.Y., Li J., Liu Y.Q., Tang B.X., Chi Z.H. (2024). Disitamab Vedotin plus Toripalimab in Patients with Locally Advanced or Metastatic Urothelial Carcinoma (RC48-C014): A Phase 1b/2 Dose-Escalation and Dose-Expansion Study. Ann. Oncol..

[19] Galsky M.D., Del Conte G., Foti S., Yu E.Y., Machiels J.-P.H., Doger B., Necchi A., De Braud F.G., Hamilton E.P., Hennequin A. (2022). Primary Analysis from DS8201-A-U105: A Phase 1b, Two-Part, Open-Label Study of Trastuzumab Deruxtecan (T-DXd) with Nivolumab (Nivo) in Patients (Pts) with HER2-Expressing Urothelial Carcinoma (UC). J. Clin. Oncol..

[20] Vlachou E., Matoso A., McConkey D., Jing Y., Johnson B.A., Hahn N.M., Hoffman-Censits J. (2023). Enfortumab Vedotin-Related Cutaneous Toxicity and Radiographic Response in Patients with Urothelial Cancer: A Single-Center Experience and Review of the Literature. Eur. Urol. Open Sci..

[21] Vlachou E., Johnson B.A., McConkey D., Jing Y., Matoso A., Hahn N.M., Hoffman-Censits J. (2024). Enfortumab Vedotin-Related Cutaneous Toxicity Correlates with Overall Survival in Patients with Urothelial Cancer: A Retrospective Experience. Front. Oncol..

[22] Lacouture M.E., Patel A.B., Rosenberg J.E., O’Donnell P.H. (2022). Management of Dermatologic Events Associated With the Nectin-4-Directed Antibody-Drug Conjugate Enfortumab Vedotin. Oncologist.

[23] Chu C.E., Sjöström M., Egusa E.A., Gibb E.A., Badura M.L., Zhu J., Koshkin V.S., Stohr B.A., Meng M.V., Pruthi R.S. (2021). Heterogeneity in NECTIN4 Expression Across Molecular Subtypes of Urothelial Cancer Mediates Sensitivity to Enfortumab Vedotin. Clin. Cancer Res. Off. J. Am. Assoc. Cancer Res..

[24] Rosenberg J., Sridhar S.S., Zhang J., Smith D., Ruether D., Flaig T.W., Baranda J., Lang J., Plimack E.R., Sangha R. (2020). EV-101: A Phase I Study of Single-Agent Enfortumab Vedotin in Patients With Nectin-4–Positive Solid Tumors, Including Metastatic Urothelial Carcinoma. J. Clin. Oncol..

[25] Klümper N., Ralser D.J., Ellinger J., Roghmann F., Albrecht J., Below E., Alajati A., Sikic D., Breyer J., Bolenz C. (2023). Membranous NECTIN-4 Expression Frequently Decreases during Metastatic Spread of Urothelial Carcinoma and Is Associated with Enfortumab Vedotin Resistance. Clin. Cancer Res..

[26] Klümper N., Tran N.K., Zschäbitz S., Hahn O., Büttner T., Roghmann F., Bolenz C., Zengerling F., Schwab C., Nagy D. (2024). NECTIN4 Amplification Is Frequent in Solid Tumors and Predicts Enfortumab Vedotin Response in Metastatic Urothelial Cancer. J. Clin. Oncol..

[27] Flaig T.W., Rosenberg J.E., Hoimes C.J., O’Donnell P.H., Mar N., Gourdin T.S., Henry S., Bilen M.A., George S., Barata P.C. (2023). Study EV-103: Neoadjuvant Treatment with Enfortumab Vedotin Monotherapy in Cisplatin-Ineligible Patients (Pts) with Muscle Invasive Bladder Cancer (MIBC): Updated Results for Cohort H. J. Clin. Oncol..

[28] Sridhar S., O’Donnell P.H., Flaig T.W., Rosenberg J.E., Hoimes C.J., Milowsky M.I., Srinivas S., George S., McKay R.R., Petrylak D.P. (2023). 2365MO Study EV-103 Cohort L: Perioperative Treatment w/Enfortumab Vedotin (EV) Monotherapy in Cisplatin (Cis)-Ineligible Patients (Pts) w/Muscle Invasive Bladder Cancer (MIBC). Ann. Oncol..

[29] Hoimes C.J., Loriot Y., Bedke J., Nishiyama H., Kataria R.S., Homet Moreno B., Galsky M.D. (2023). Perioperative Enfortumab Vedotin (EV) plus Pembrolizumab (Pembro) versus Chemotherapy in Cisplatin-Eligible Patients (Pts) with Muscle-Invasive Bladder Cancer (MIBC): Phase 3 KEYNOTE-B15/EV-304. J. Clin. Oncol..

[30] Powles T., Catto J.W.F., Galsky M.D., Al-Ahmadie H., Meeks J.J., Nishiyama H., Vu T.Q., Antonuzzo L., Wiechno P., Atduev V. (2024). Perioperative Durvalumab with Neoadjuvant Chemotherapy in Operable Bladder Cancer. N. Engl. J. Med..

[31] Bajorin D.F., Witjes J.A., Gschwend J.E., Schenker M., Valderrama B.P., Tomita Y., Bamias A., Lebret T., Shariat S.F., Park S.H. (2021). Adjuvant Nivolumab versus Placebo in Muscle-Invasive Urothelial Carcinoma. N. Engl. J. Med..

[32] A Phase 3, Randomized, Open-Label Study to Evaluate Perioperative Enfortumab Vedotin Plus Pembrolizumab (MK-3475) Versus Neoadjuvant Gemcitabine and Cisplatin in Cisplatin-Eligible Participants with Muscle-Invasive Bladder Cancer (MK-3475-B15/KEYNOTE-B15/EV-304). https://clinicaltrials.gov/study/NCT04700124.

[33] Galsky M.D., Necchi A., Shore N.D., Plimack E.R., Jia C., Sbar E., Homet Moreno B., Witjes J.A. (2021). KEYNOTE-905/EV-303: Perioperative Pembrolizumab or Pembrolizumab plus Enfortumab Vedotin (EV) and Cystectomy Compared to Cystectomy Alone in Cisplatin-Ineligible Patients with Muscle-Invasive Bladder Cancer (MIBC). J. Clin. Oncol..

[34] A Randomized Phase 3 Study Evaluating Cystectomy With Perioperative Pembrolizumab and Cystectomy With Perioperative Enfortumab Vedotin and Pembrolizumab Versus Cystectomy Alone in Participants Who Are Cisplatin-Ineligible or Decline Cisplatin with Muscle-Invasive Bladder Cancer (KEYNOTE-905/EV-303). https://clinicaltrials.gov/study/NCT03924895.

[35] Powles T., Drakaki A., Teoh J.Y.-C., Grande E., Fontes-Sousa M., Porta C., Wu E., Goluboff E.T., Ho S., Hois S. (2022). A Phase 3, Randomized, Open-Label, Multicenter, Global Study of the Efficacy and Safety of Durvalumab (D) + Tremelimumab (T) + Enfortumab Vedotin (EV) or D + EV for Neoadjuvant Treatment in Cisplatin-Ineligible Muscle-Invasive Bladder Cancer (MIBC) (VOLGA). J. Clin. Oncol..

[36] A Phase III Randomized, Open-Label, Multicenter Study to Determine the Efficacy and Safety of Durvalumab in Combination With Tremelimumab and Enfortumab Vedotin or Durvalumab in Combination With Enfortumab Vedotin for Perioperative Treatment in Patients Ineligible for Cisplatin or Who Refuse Cisplatin Undergoing Radical Cystectomy for Muscle Invasive Bladder Cancer (VOLGA). https://clinicaltrials.gov/study/NCT04960709.

[37] Enfortumab Vedotin in Combination with Pembrolizumab for Locally Advanced and/or Node Positive Urothelial Carcinoma Prior to Surgery (EV-ECLIPSE). https://clinicaltrials.gov/study/NCT05239624.

[38] A Phase 1/2 Study of V940 Plus Pembrolizumab with or Without Enfortumab Vedotin in Muscle-Invasive Urothelial Carcinoma (MIUC) (INTerpath-005). https://clinicaltrials.gov/study/NCT06305767.

[39] Phase Ib/II Study of Enfortumab Vedotin and Pembrolizumab Combined with Radiotherapy as a Bladder-Sparing Trimodality Therapy in Muscle Invasive Bladder Cancer. https://clinicaltrials.gov/study/NCT06470282.

[40] Kamat A.M., Steinberg G.D., Inman B.A., Kates M.R., Uchio E.M., Porten S.P., Roupret M., Redorta J., Catto J.W.F., Kulkarni G.S. (2023). Study EV-104: Phase 1 Study of Intravesical Enfortumab Vedotin for Treatment of Patients with Non-Muscle Invasive Bladder Cancer (NMIBC)—Trial in Progress. J. Clin. Oncol..

[41] Balar A.V., Kamat A.M., Kulkarni G.S., Uchio E.M., Boormans J.L., Roumiguié M., Krieger L.E.M., Singer E.A., Bajorin D.F., Grivas P. (2021). Pembrolizumab Monotherapy for the Treatment of High-Risk Non-Muscle-Invasive Bladder Cancer Unresponsive to BCG (KEYNOTE-057): An Open-Label, Single-Arm, Multicentre, Phase 2 Study. Lancet Oncol..

[42] Narayan V.M., Boorjian S.A., Alemozaffar M., Konety B.R., Shore N.D., Gomella L.G., Kamat A.M., Bivalacqua T.J., Montgomery J.S., Lerner S.P. (2024). Efficacy of Intravesical Nadofaragene Firadenovec for Patients With Bacillus Calmette-Guérin-Unresponsive Nonmuscle-Invasive Bladder Cancer: 5-Year Follow-Up From a Phase 3 Trial. J. Urol..

[43] Chamie K., Chang S.S., Kramolowsky E., Gonzalgo M.L., Agarwal P.K., Bassett J.C., Bjurlin M., Cher M.L., Clark W., Cowan B.E. (2022). IL-15 Superagonist NAI in BCG-Unresponsive Non–Muscle-Invasive Bladder Cancer. NEJM Evid..

[44] FDA Approves Pembrolizumab for BCG-Unresponsive, High-Risk Non-Muscle Invasive Bladder Cancer. https://www.fda.gov/drugs/resources-information-approved-drugs/fda-approves-pembrolizumab-bcg-unresponsive-high-risk-non-muscle-invasive-bladder-cancer.

[45] FDA Approves First Adenoviral Vector-Based Gene Therapy for High-Risk Bacillus Calmette-Guérin Unresponsive Non-Muscle Invasive Bladder Cancer. https://www.fda.gov/drugs/resources-information-approved-drugs/fda-approves-first-adenoviral-vector-based-gene-therapy-high-risk-bacillus-calmette-guerin.

[46] FDA Approves Nogapendekin Alfa Inbakicept-Pmln for BCG-Unresponsive Non-Muscle Invasive Bladder Cancer. https://www.fda.gov/drugs/resources-information-approved-drugs/fda-approves-nogapendekin-alfa-inbakicept-pmln-bcg-unresponsive-non-muscle-invasive-bladder-cancer.

[47] A Phase II, Open-Label, Single-Arm, Multi-Center Study of Neoadjuvant Enfortumab Vedotin and Pembrolizumab in Cisplatin-Eligible Upper Tract Urothelial Cancer (NEPTUNE). https://clinicaltrials.gov/study/NCT06356155.

[48] Neoadjuvant Enfortumab Vedotin in High-Grade Urothelial Carcinoma of the Upper Urinary Tract (Including Ureter and Renal Pelvis). https://clinicaltrials.gov/study/NCT05868265.

[49] Li K., Zhou Y., Zang M., Jin X., Li X. (2024). Therapeutic Prospects of Nectin-4 in Cancer: Applications and Value. Front. Oncol..

[50] Khosravanian M.J., Mirzaei Y., Mer A.H., Keyhani-Khankahdani M., Abdinia F.S., Misamogooe F., Amirkhani Z., Bagheri N., Meyfour A., Jahandideh S. (2024). Nectin-4-Directed Antibody-Drug Conjugates (ADCs): Spotlight on Preclinical and Clinical Evidence. Life Sci..

[51] Zhou W., Fang P., Yu D., Ren H., You M., Yin L., Mei F., Zhu H., Wang Z., Xu H. (2023). Preclinical Evaluation of 9MW2821, a Site-Specific Monomethyl Auristatin E–Based Antibody–Drug Conjugate for Treatment of Nectin-4–Expressing Cancers. Mol. Cancer Ther..

[52] Ye D.-W., Zhang J., Yang H., Yang J., Zheng T., Sun H., Wan X., Lan G., Sun G., Zhang X. (2024). Phase 1 Dose Escalation of SYS6002 (CRB-701), a next-Generation Nectin-4 Targeting Antibody Drug Conjugate (ADC). J. Clin. Oncol..

[53] Rosenberg J., Sabatier R., Viceneux A., de Rouge T.L.M., Champiat S., Lebellec L., Barthélémy P., Sonpavde G., Gao X., Niglio S. (2024). Abstract CT084: A Phase 1 Study of LY4101174 (ETx-22), an Antibody-Drug Conjugate Targeting Nectin-4, in Patients with Advanced or Metastatic Urothelial Cancer and Other Solid Tumors (Trial in Progress). Cancer Res..

[54] Jiang L., Song Z., Gong Y., Jin J., Ding Y., Tang L., Deng X., Li X., Li S., Cheng X. (2024). A Phase 1 Study of BAT8007, an Anti-Nectin-4 Monoclonal Antibody-Exatecan Conjugate, in Patients with Advanced Solid Tumors. J. Clin. Oncol..

[55] Shahmoradgoli M., Hau A., Lee D.J., Wang A., Challita P.P., Betancourt O., Sisson W., Kuo M.M., Zhang K., Goldson A. (2024). Abstract 1902: ADRX-0706 Nectin-4 Antibody-Drug Conjugate PK/PD Characterization Elucidates Its Widened Therapeutic Window. Cancer Res..

[56] Sagar D., Srinivasan M., Lindquist K., Guo Q., Wong W., Lebron M.B., Sattler R.M., Zhou J., Helms W., Boyles J. (2024). Abstract 1872: A next Generation Treatment for Nectin-4 Positive Cancers—Preclinical Characterization of LY4052031, an Anti-Nectin-4 Antibody, Conjugated to a Novel Camptothecin Payload. Cancer Res..

[57] Tang B., Sheng X., Guo J., Niu H., Shen Y., Jiang S., Fu B., Guo J., Wahafu W., Yao K. (2025). Nectin-4 Targeted ADC, SHR-A2102, in Patients with Advanced or Metastatic Urothelial Carcinoma: A Phase 1 Study. J. Clin. Oncol..

[58] Wang J., Xing C., Liu H., Cugnetti A.P.G., Wheeler C., Lucas M., Frey G., Chang C., Boyle W.J., Short J.M. (2020). Abstract 4560: Conditionally Active Biologics (CAB): A Novel Class of Molecules Targeting Solid Tumors. Cancer Res..

[59] Frey G., Wang J., Johnson K., Liu H., Wheeler C., Xing C., Cugnetti A.P., McNeeley P., Joyner S., Chang C. (2023). Abstract 1541: NextGen Conditionally Active Biologic (CAB) Anti-Nectin-4-ADC with Improved Stability and Safety. Cancer Res..

[60] BioAtla Announces FDA Clearance of Investigational New Drug Application for BA3361, a CAB-Nectin-4 Antibody Drug Conjugate for the Treatment of Multiple Tumors. https://www.globenewswire.com/news-release/2024/05/06/2875781/0/en/BioAtla-Announces-FDA-Clearance-of-Investigational-New-Drug-Application-for-BA3361-a-CAB-Nectin-4-Antibody-Drug-Conjugate-for-the-Treatment-of-Multiple-Tumors.html.

[61] Rigby M., Bennett G., Chen L., Mudd G.E., Harrison H., Beswick P.J., Van Rietschoten K., Watcham S.M., Scott H.S., Brown A.N. (2022). BT8009; A Nectin-4 Targeting Bicycle Toxin Conjugate for Treatment of Solid Tumors. Mol. Cancer Ther..

[62] Hurov K., Lahdenranta J., Upadhyaya P., Haines E., Cohen H., Repash E., Kanakia D., Ma J., Kristensson J., You F. (2021). BT7480, a Novel Fully Synthetic Bicycle Tumor-Targeted Immune Cell Agonist^TM^ (Bicycle TICA^TM^) Induces Tumor Localized CD137 Agonism. J. Immunother. Cancer.

[63] Yan S., Zhang G., Luo W., Xu M., Peng R., Du Z., Liu Y., Bai Z., Xiao X., Qin S. (2024). PROTAC Technology: From Drug Development to Probe Technology for Target Deconvolution. Eur. J. Med. Chem..

